# The physical and mental health of Australian truck drivers: a national cross-sectional study

**DOI:** 10.1186/s12889-022-12850-5

**Published:** 2022-03-08

**Authors:** Caryn van Vreden, Ting Xia, Alex Collie, Elizabeth Pritchard, Sharon Newnam, Dan I. Lubman, Abilio de Almeida Neto, Ross Iles

**Affiliations:** 1 Healthy Working Lives Research Group, School of Public Health and Preventive Medicine, Monash University, Level 2, 553 St Kilda Road, Melbourne, 3004 Australia; 2 Accident Research Centre, Monash University, Melbourne, Australia; 3 Turning Point, Eastern Health and Monash Addiction Research Centre, Eastern Health Clinical School, Monash University, Melbourne, Australia; 4 Centre for Work Health and Safety, New South Wales Government, Sydney, Australia

## Abstract

**Background:**

The negative health consequences of truck driving are well documented. However, despite the distinct occupational challenges between long- and short-haul driving, limited research has been conducted on how the health profile of these drivers differ. The aims of this study were to characterise the physical and mental health of Australian truck drivers overall, and to identify any differences in factors influencing the health profile of long-haul compared to short-haul drivers.

**Design, setting, and participants:**

In this cross-sectional study, 1390 Australian truck drivers completed an online survey between August 2019 and May 2020. Questions included validated measures of psychological distress, general health, work ability and health-related quality-of-life. Participants driving 500 km or more per day were categorised as long-haul and those driving less than 500 km as short-haul.

**Results:**

The majority of survey respondents were classified as either overweight (25.2%) or obese (54.3%). Three in ten reported three or more chronic health conditions (29.5%) and poor general health (29.9%). The most commonly diagnosed conditions were back problems (34.5%), high blood pressure (25.8%) and mental health problems (19.4%). Chronic pain was reported by 44% of drivers. Half of drivers reported low levels of psychological distress (50.0%), whereas 13.3 and 36.7% experienced severe or moderate level of psychological distress respectively. There were a small number of differences between the health of long- and short-haul drivers. A higher proportion of short-haul drivers reported severe psychological distress compared to long-haul drivers (15.2% vs 10.4%, χ^2^ = 8.8, 0.012). Long-haul drivers were more likely to be obese (63.0% vs 50.9%, χ2 = 19.8, < 0.001) and report pain lasting over a year (40.0% vs 31.5%, χ^2^ = 12.3, 0.006). Having more than one diagnosed chronic condition was associated with poor mental and physical health outcomes in both long- and short-haul drivers.

**Conclusion:**

Australian truck drivers report a high prevalence of multiple physical and mental health problems. Strategies focused on improving diet, exercise and preventing chronic conditions and psychological distress, that can also be implemented within the unique occupational environment of trucking are needed to help improve driver health. Further research is needed to explore risk and protective factors that specifically affect health in both short-haul and long-haul drivers.

**Supplementary Information:**

The online version contains supplementary material available at 10.1186/s12889-022-12850-5.

## Introduction

Road freight transport is essential for global economies, with an estimated 18% of goods transported across the world in 2015 occurring by road [1]. In Australia, approximately 75% of non-bulk domestic freight is carried on the road [2]. Truck driving is also the most common occupation among Australian males [3], but the industry is currently facing severe driver shortages, high levels of ill-health within a rapidly aging workforce and difficulty recruiting younger drivers [4, 5]. With the demand for global freight estimated to triple by 2050 [1], it is crucial for the transport industry to invest in their workers and address the health and lifestyle risk factors associated with truck driving. Improving the health of the workforce will benefit drivers directly, but also help to ensure the future stability of road freight transport.

The risk factors associated with being a professional truck driver are well established [6, 7]. The sedentary nature of the occupation, heavily regulated work and rest times, irregular sleep patterns, limited opportunity for exercise or access to nutritious food on the road, all contribute to an increased risk of multiple health conditions [8–10]. Back pain, hypertension, obesity, sleep apnoea, diabetes and depression have all been shown to be common among this group [7]. It has been reported that transport workers are at increased risk of work-related injury [11], and that crashes only account for 17% of the burden of injury and illness among truck drivers [12]. Cardiovascular disease has been identified as the most common cause of death among transport workers, whilst younger drivers are at greater risk of suicide [13]. Furthermore, patterns of medication use and infrequent access of mental health services suggest that drivers do not always receive evidence-based care following recommended guidelines [14, 15], further compounding the negative impact of pre-existing health conditions.

It is important to note that truck driving is not a homogenous profession. Drivers operating over long distances (also known as long-haul drivers) and those operating over shorter average daily distances (short-haul drivers) are exposed to a different set of occupational demands, working conditions, personal environments and risk factors. Long-haul drivers are more likely to be exposed to long periods of isolated and sedentary work hours, poor sleep hygiene patterns and lack of access to nutritious food on the road [9, 10]. Short-haul drivers spend more time driving in high density traffic and may experience greater time pressures to make multiple deliveries in a day [8]. Factors within the personal environment, such as relationships with family and friends, also impacts on the health and wellbeing of these populations. For instance, long-haul drivers spend many nights away from their family potentially leading to breakdown of family relationships [16]. Most prior research on driver health has focused on long-haul drivers, with few studies examining the health of short-haul drivers. In order to determine the value of tailored approaches to intervention design between these occupational roles, the industry requires greater knowledge around how work factors differentially affect the health of long- and short-haul drivers.

To date, the majority of existing research into the physical and mental health of truck drivers has been conducted outside of Australia [7], while large studies conducted within Australia have focused almost exclusively on specific health conditions, like obesity and mental health conditions, [17–19] or the safety of drivers [9, 20, 21]. While Australia has a land mass similar to the US and larger than much of Europe, most of Australia’s population is concentrated on the east coast, impacting how freight is moved across the country. Non-uniform regulations across states, as well as ongoing legislative changes means there is a unique overlay of work health and safety laws in Australia under which drivers must operate [21]. Any intervention to improve the health and wellbeing of drivers must be implemented in the Australian context, so it is essential to understand the specific health challenges Australian drivers face. There is limited research on the health profile of Australian truck drivers overall, and health differences between long- and short-haul drivers are unknown. As such, the aim of this paper is to a) characterise the health profile of Australian truck drivers b) compare the health profiles of long- and short-haul drivers; and c) determine if there are differences in the factors influencing health outcomes of long- versus short-haul truck drivers.

## Methods

### Data collection and recruitment

This cross-sectional online survey was administered via the Qualtrics Insight Platform [22] and was designed to be completed within 10 min. Truck drivers, particularly long-haul drivers, are a hard to reach population for research, so it was essential that the survey was brief, contained language drivers would recognise and understand, and drivers could choose to participate and remain completely anonymous. Recruitment occurred through a multi-pronged sampling approach designed to reach drivers as directly as possible, including targeted social media posts, industry specific publications and study partner internal communications with employees and union members. Study partners included a large national private transport company operating a fleet of more than 5000 vehicles, the peak national transport workers’ union representing over 70,000 Australian transport workers, and a state government work health and safety department. Targeted paid advertisements on Facebook were also used to capture a broad distribution of ages and work types.

Drivers were eligible to participate if they were: a) Employed in a job involving the transport of goods in the 12 months prior to the survey, and b) Drove a vehicle (van or larger) for their job (> 4.5 t). Drivers must have been able to complete the survey in English.

### Measurement

The online survey was developed by the research team with input from representatives of the transport industry. The survey (Additional file 1) included items that addressed health outcomes and their determinants as identified in a systematic review of health in truck drivers [23]. Where possible, items were selected to include scales also used in the Australian National Health Survey [24]. The survey was piloted with a group of experienced drivers (*n* = 5) recruited by a private transport company and national transport workers’ union, to ensure the content was appropriate. See Table 1 for a summary of the information captured in the online survey**.**

**Table 1:** Information captured in the survey

Personal characteristics	Work characteristics	Health Profile
Age Sex Height Weight	Work type Employment type Experience Payment type Working hours Shift type Vehicle type Working for > 1 company	Number of diagnosed conditions [24] Psychological distress [25] General Health [26] Pain intensity [27] Pain duration Health-related quality of life (HRQOL) – utility score [28] HRQOL - VAS score Work Ability [29]

#### Personal and work characteristics

Personal and work characteristics were captured to provide information on the study cohort and allow characterisation of work type into long- (driving ≥500 km/shift) and short-haul (driving < 500 km/shift) drivers. This definition of long- and short-haul driving was tested with partners and drivers before being implemented in the survey. Work characteristics included questions about working conditions and factors specific to driving (i.e., driving experience, typical driving distance/shift, employment type, payment type, shift type, working hours and type of vehicle driven).

#### Health profile

The health profile of drivers was established through questions on health conditions and other factors known to influence overall health based on current evidence. This included an estimate of height and weight for Body Mass Index (BMI) calculation, which was grouped into three categories; under or normal weight (BMI < 25), overweight (BMI = 25–29.9), or obese (BMI ≥ 30) [30]. A list of specific chronic conditions were included, derived from the Australian National Health survey [24] and previous Driving Health Study reports [12, 13, 31], asking drivers to identify which conditions they had been diagnosed with by a health professional. The number of conditions for each driver was then summed to identify drivers with multiple health conditions.

Psychological distress was measured using the 6-item Kessler Psychological Distress Scale (K6) [25]. This validated scale includes six questions converted to a scale from 0 to 24 and categorised as none or low (score of 0–4), moderate (score of 5–12) or severe (score of ≥13) psychological distress [32]. Self-rated general health was measured with the first question of the Short-Form 12 [26] and described as Excellent/Very good, Good, and Poor/Fair [33]. Pain duration and intensity were derived from items 1 and 2 of the Örebro Musculoskeletal Pain Questionnaire [27]. Pain intensity was determined on a scale of 0 (no pain)-10 (worst possible pain) and converted into four categories: No pain (0), Mild (1–3), Moderate (4–6) and Severe (7–10). Pain duration was defined as either < 3 months, 3–12 months or > 12 months, with both latter categories indicating chronic pain [34]. The 5-level EuroQol (EQ-5D-5L) questionnaire [35] was used to determine the Health-related quality of life (HRQOL) utility scores and EQ-visual analogue scale (VAS) rating. HRQOL scores were calculated using a scoring algorithm developed by Devlin et al. [28], selected due to the normative values for an Australian population being available [36]. Potential values ranged from − 0.281 to 1 with lower scores representing progressively poorer health and negative values considered states worse than death. The EQ-VAS provided a rating of self-perceived health scored from 0 (worst possible health) to 100 (best possible health). Work ability was measured using the first item of the Work Ability Index [29], asking participants to describe their ability to work on a scale of 0 (completely unable to work) to 10 (able to work at their best) and categorised as poor (0–5), moderate (6–7), good (8–9) and excellent (10).

### Data analysis

Data cleaning and analyses were conducted using IBM SPSS Statistics for Windows, V26 [37]. Variables with groups < 20 were combined with larger categories or collapsed into “other” categories. Participants with missing responses in key questions (*n* = 416), such as work type were removed from analyses. Missing values or “Prefer not to say” responses comprised < 3% of the remaining items and were therefore not included in results.

Counts and percentages were used to summarise the survey data. The characteristics of long- and short-haul drivers were reported separately to enable comparisons. The Chi^2^ statistic was used to determine statistical significance between group proportions. The HRQOL utility score and EQ-VAS mean score was compared using independent t-tests.

In order to establish differences in the factors influencing mental and physical outcomes for long- versus short-haul truck drivers, psychological distress and HRQOL were chosen as dependent variables for further regression analysis. Ordinal logistic modelling was used to examine predictors of severe psychological distress, and generalised linear regression modelling was used to examine predictors of decreased HRQOL.

HRQOL utility score was converted to a *disutility score* (1-HRQOL score) and log transformed (log (*disutility score*) + 1) to be entered as the dependent variable in linear regression model. An increase in *Disutility score* was indicative of a decreased HRQOL and worse health outcome. Independent variables entered into both models included: age, employment type, working> 1 company, shift type, vehicle type, payment type, working hours, BMI and diagnosed conditions. The measure of effect was reported in Odds Ratio (OR) for ordinal logistic models, and in exponentiated coefficient (Exp(β) for linear models. Statistical significance was set at *p* < 0.05.

## Results

### Personal and work characteristics of survey respondents

**Fig. 1:**
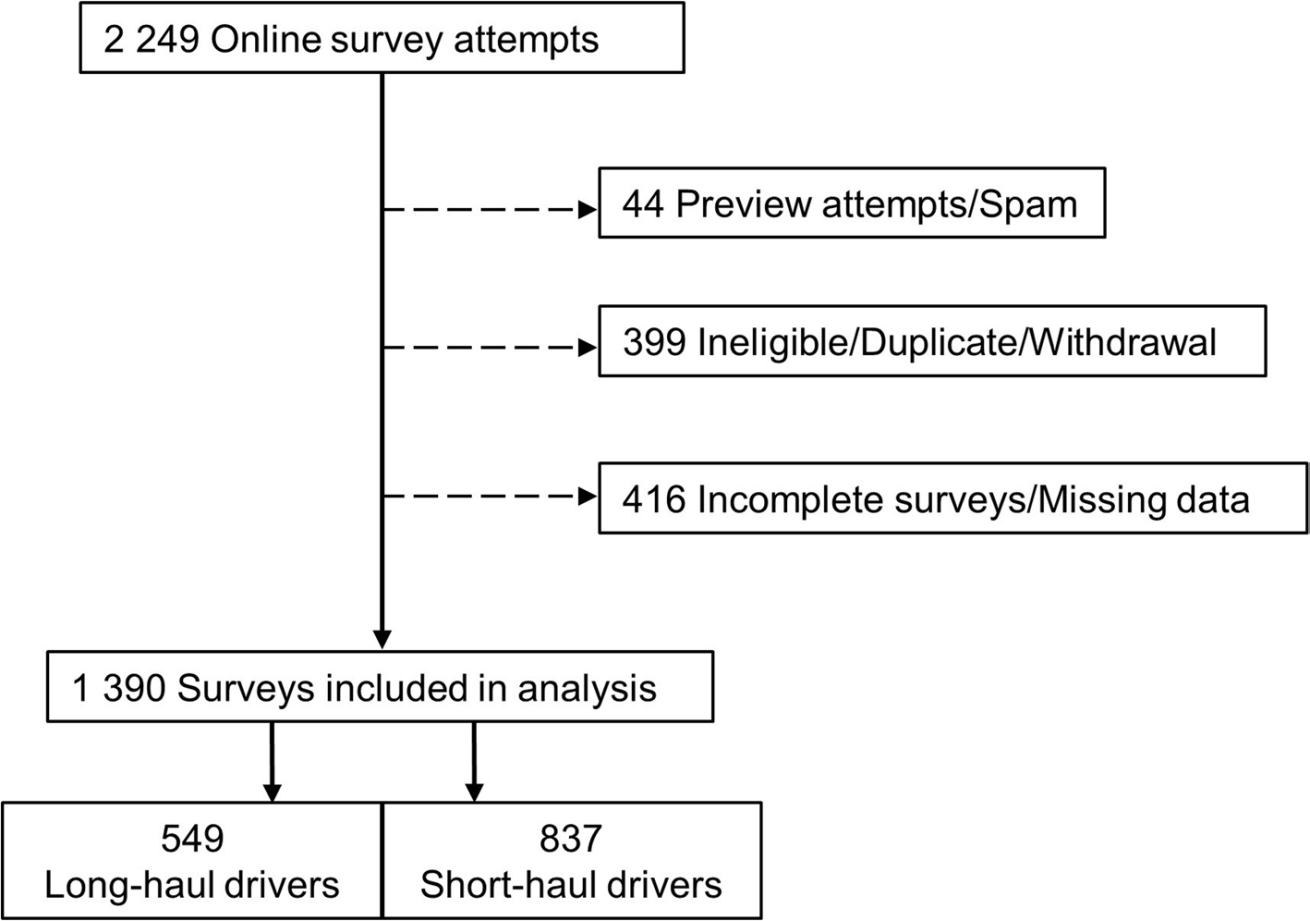
Flow chart of survey responses

The final sample for analysis consisted of 1390 respondents with completed surveys (Fig. 1), with 39.5% driving long-haul and 60.2% short-haul. The personal and work characteristics of the truck drivers completing the survey can be found in Table 2. The majority of respondents were male (97.1%) and evenly spread across age groups. Both gender and age distribution was consistent with Australian labour force data indicating that this sample is representative of the driving workforce [3].

**Table 2:** Personal and work characteristics of survey respondents

	Whole cohort *N* = 1390		Long-haul *N* = 549 (39.5%)		Short-haul *N* = 837 (60.2%)			
	*n*	%	*n*	%	*n*	%	Χ^**2**^ (df)	*p*
Age								
< 35 years	367	26.4%	141	25.7%	225	26.9%	4.3 (3)	0.231
35–44 years	273	19.6%	95	17.3%	177	21.2%		
45–55 years	364	26.2%	153	27.9%	210	25.1%		
> 55 years	383	27.6%	159	29.0%	223	26.7%		
Sex								
Male	1349	97.1%	531	97.4%	814	97.7%	0.1 (1)	0.231
Female	33	2.4%	14	2.6%	19	2.3%		
Employment type								
Owner driver	190	13.7%	82	15.1%	107	12.9%	1.4 (1)	0.242
Employee driver	1181	85.5%	461	84.9%	724	87.2%		
Experience								
< 5 years	283	20.4%	91	16.6%	191	22.8%	11.9 (2)	**0.003**
5–20 years	533	38.3%	204	37.2%	328	39.2%		
> 20 years	573	41.2%	253	46.2%	318	38.0%		
Payment type								
Flat rate	459	33.0%	123	22.7%	335	40.4%	520.9 (4)	**< 0.001**
Single time pay	417	30.0%	48	8.8%	368	44.3%		
Kilometre rate	285	20.5%	263	48.4%	22	2.7%		
Per trip/delivery	129	9.3%	77	14.2%	52	6.3%		
Other	86	6.2%	32	5.9%	53	6.4%		
Working hours/week								
≤ 40	156	11.2%	38	7.0%	117	14.1%	169.5 (2)	**< 0.001**
41–60	700	50.4%	185	34.1%	513	61.8%		
> 60	521	37.5%	320	58.9%	200	24.1%		
Mean (SD)	60.62 (17.77)		69.34 (18.83)		55.59 (15.06)			
Shift type								
Multiple trips between same location	789	56.8%	140	25.7%	647	78.0%	423.4 (2)	**< 0.001**
Single trip between 2 locations	334	24.0%	274	50.3%	60	7.2%		
Multiple trips between 2 locations	255	18.3%	131	24.0%	122	14.7%		
Vehicle type								
B double	451	32.4%	279	51.0%	171	20.5%	289.8 (3)	**< 0.001**
Articulated truck	432	31.1%	126	23.0%	305	36.5%		
Rigid truck and other	349	25.1%	37	6.8%	311	37.2%		
Road train	153	11.0%	105	19.2%	48	5.7%		
Working for more than one company								
Yes	183	13.2%	89	16.3%	93	11.2%	7.5 (1)	**0.006**
No	1197	86.1%	457	83.7%	737	88.8%		

Most drivers (85.5%) were employee drivers and 13.7% identified as owner drivers. This was comparable to Australian labour force data reporting nearly 14% of transport workers working as independent contractors [3]. Professional experience driving a truck ranged from > 20 years (41.2%), to < 5 years (20.4%). Half of the drivers (50.4%) reported working between 41 and 60 h/week. The most common working shift was multiple trips between the same location or “home base” (56.8%), followed by a long single trip between two locations (24.0%).

#### Long-haul versus short-haul truck drivers

Chi-square tests showed no significant differences in sex, age or employment type between long- and short-haul drivers (Table 2). However, a greater proportion of long-haul drivers had > 20 years’ experience, whereas more short-haul drivers had < 5 years’ experience (χ^2^(2) = 11.9, *p* = 0.003) (Table 2). More long-haul drivers reported working > 60 h/week compared to short-haul drivers (58.9% vs 24.1%, (χ^2^(2) = 169.5, *p* < 0.001). Half of the long-haul drivers had shifts of a single long trip (50.3%), whereas the great majority of short-haul drivers took multiple trips between the same location (78.0%) per shift (χ^2^(2) = 423.4, *p* < 0.001). Vehicle type also differed between long- and short-haul drivers (χ^2^(3) = 289.8, *p* < 0.001).

### Health profile of survey respondents

**Fig. 2:**
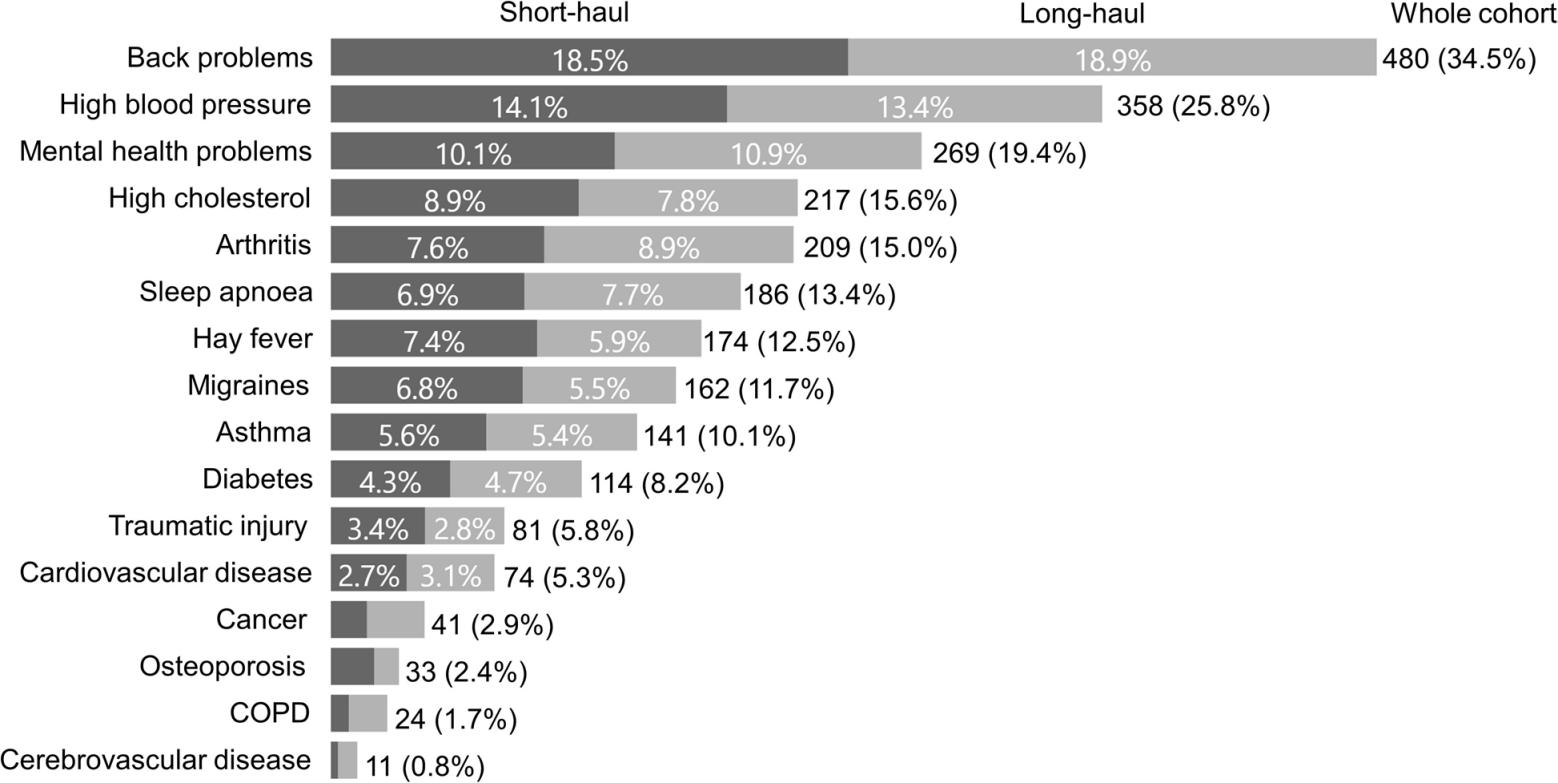
Diagnosed health conditions by work type

The majority of drivers were classified as overweight or obese (79.5%) (Table 3). The most commonly reported conditions were back problems, high blood pressure and mental ill-health (e.g. depression and anxiety) (Fig. 2). Most drivers reported being diagnosed with at least one listed condition, with 29.5% reporting more than two.

**Table 3:** Health profile of survey respondents

	Whole cohort *N* = 1390		Long-haul *N* = 549 (39.5%)		Short-haul *N* = 837 (60.2%)			
	*n*	%	*n*	%	*n*	%	Χ^**2**^ (df)	*p*
Body Mass Index								
Under or normal weight	252	18.1%	79	14.8%	173	21.1%	19.8 (2)	**< 0.001**
Overweight	350	25.2%	119	22.2%	229	28.0%		
Obese	755	54.3%	337	63.0%	416	50.9%		
BMI- mean (SD)	31.95 (6.99)		33.10 (7.47)		31.19 (6.56)			
Number of diagnosed health conditions								
No conditions	346	24.9%	133	24.7%	212	25.7%	0.7 (3)	0.885
1 condition	364	26.2%	139	25.8%	222	26.9%		
2 conditions	248	17.8%	102	18.9%	146	17.7%		
≥ 3 conditions	410	29.5%	165	30.6%	245	29.7%		
Mean (SD)	1.85 (1.85)		1.86 (1.87)		1.85 (1.85)			
Psychological distress								
None or low	695	50.0%	296	53.9%	397	47.4%	8.8 (2)	**0.012**
Moderate	510	36.7%	196	35.7%	313	37.4%		
Severe	185	13.3%	57	10.4%	127	15.2%		
General Health								
Very good/Excellent	437	31.4%	169	30.8%	268	32.0%	0.3 (2)	0.851
Good	538	38.7%	212	38.6%	323	38.6%		
Poor/Fair	415	29.9%	168	30.6%	246	29.4%		
Pain - intensity								
None	523	37.6%	198	36.1%	324	38.7%	4.9 (3)	0.183
Mild	321	23.1%	116	21.1%	203	24.3%		
Moderate	398	28.6%	172	31.3%	225	26.9%		
Severe	148	10.6%	63	11.5%	85	10.2%		
Pain – duration								
< 3 months	245	17.6%	80	14.7%	164	19.7%	12.3 (2)	**0.006**
3–12 months	131	9.4%	50	9.2%	81	9.7%		
> 12 months	482	34.7%	218	40.0%	262	31.5%		
Work ability								
Poor	200	14.4%	85	15.5%	115	13.7%	3.4 (3)	0.334
Moderate	232	16.7%	82	14.9%	148	17.7%		
Good	565	40.6%	217	39.5%	346	41.3%		
Excellent	393	28.3%	165	30.1%	228	27.2%		
Health related quality of life	**mean**	**SD**	**mean**	**SD**	**mean**	**SD**	**t**	**p**
– utility score -	0.83	0.16	0.83	0.17	0.84	0.16	0.7	0.7
EQ-VAS scale score	72.2	19.8	72	20.5	72.4	19.4	0.4	0.209

Half of the drivers had no or low psychological distress (50.0%), whereas 13.3 and 36.7% were experiencing severe and moderate psychological distress respectively (Table 3). Approximately two thirds of drivers reported being in either excellent, very good or good (70.1%) health. Around two thirds (62.3%) reported pain in the week prior to the survey, with 10.6% describing severe pain. Over a third of the drivers surveyed (44.1%) had experienced chronic pain (lasting longer than 3 months), representing 71.4% of those reporting pain. The mean (SD) of HRQOL utility score for all truck drivers was 0.83 (0.16) (Table 3), with a mean (SD) EQ-VAS score of 72.2 (19.9).

#### Health profile of long- versus short-haul drivers

Chi-square tests revealed significant differences between long- and short-haul driver BMI, level of psychological distress and pain duration. Obesity was more common among long-haul drivers (63.0% vs 50.9%) (χ^2^(2) = 19.8, *p* < 0.001). A greater percentage of short-haul drivers had severe psychological distress compared to long-haul drivers (15.2% vs 10.4%, χ^2^(2) = 8.8, *p* = 0.012). A larger proportion of long-haul drivers reported pain lasting > 12 months (40.0%) compared to short-haul drivers (31.5%) (χ^2^(2) = 12.3, *p* = 0.006). There were no significant differences in the type (Fig. 2) or number of diagnosed medical conditions between long- and short-haul drivers (Table 3). Long- and short-haul drivers had comparable self-rated general health, pain severity, HRQOL utility and EQ-VAS scores.

### Determinants of health outcomes in long- and short-haul drivers

#### Psychological distress

Age and number of diagnosed conditions were associated with psychological distress in both work types (Table 4). Compared to drivers > 55 years, those < 35 had approximately 4.3 times greater odds of having severe levels of psychological distress for both long- and short-haul drivers. The impact of diagnosed conditions on psychological distress was amplified, with large increases in the odds of having severe psychological stress for both long-haul (more than 7 times) and short-haul (more than 14 times) drivers for those reporting ≥3 conditions. Short-haul drivers working ≤40 h/week had lower odds of having severe psychological distress than those working between 41 and 60 h.

**Table 4:** Results of ordinal logistic regression stratified by work type

Determinants of severe psychological distress	Long-haul drivers				Short-haul drivers			
	OR	95% CI		*p*	OR	95% CI		*p*
Age								
< 35 years	**4.27**	**2.48**	**7.36**	**< 0.001**	**4.29**	**2.85**	**6.48**	**< 0.001**
35–44 years	**2.68**	**1.48**	**4.88**	**0.001**	**1.62**	**1.06**	**2.49**	**0.027**
45–54 years	**2.33**	**1.38**	**3.91**	**0.001**	1.48	0.99	2.21	0.058
> 55 years	1				1			
Employment type								
Owner driver	1.17	0.61	2.24	0.645	0.70	0.41	1.20	0.194
Employee driver	1				1			
Work for > 1 company								
Yes	1.14	0.63	2.07	0.661	0.74	0.46	1.21	0.235
No	1				1			
Shift type								
Multiple trips between same base	1.03	0.62	1.72	0.912	0.81	0.54	1.23	0.326
Single long trip between 2 locations	0.74	0.47	1.17	0.200	0.75	0.39	1.46	0.400
Vehicle type								
Multiple trips between 2 locations	1				1			
Rigid truck and other	1.38	0.60	3.15	0.451	0.75	0.53	1.05	0.090
Road train	1.34	0.57	3.19	0.502	0.62	0.33	1.18	0.143
B Double	1.20	0.54	2.69	0.658	0.73	0.49	1.10	0.137
Articulated truck	1				1			
Payment type								
Other	1.07	0.43	2.68	0.887	1.34	0.72	2.50	0.353
Per trip/delivery	1.42	0.76	2.63	0.270	1.02	0.52	2.00	0.962
Single time pay	1.37	0.66	2.84	0.400	0.78	0.56	1.07	0.126
Km rate	1.12	0.69	1.83	0.647	1.06	0.41	2.78	0.898
Flat rate	1				1			
Working hours								
≤ 40 h	1.33	0.88	2.02	0.178	**0.57**	**0.36**	**0.89**	**0.015**
> 60 h	0.81	0.35	1.88	0.625	1.28	0.90	1.80	0.164
41–60 h	1				1			
BMI								
Obese	0.64	0.37	1.10	0.106	0.98	0.67	1.44	0.930
Overweight	0.58	0.30	1.09	0.092	1.03	0.68	1.57	0.887
Under or normal weight	1				1			
Diagnosed conditions								
1 condition	**2.89**	**1.65**	**5.06**	**< 0.001**	**2.28**	**1.50**	**3.46**	**< 0.001**
2 conditions	**4.83**	**2.64**	**8.83**	**< 0.001**	**3.22**	**2.03**	**5.11**	**< 0.001**
≥ 3 conditions	**7.41**	**4.22**	**13.02**	**< 0.001**	**9.29**	**6.01**	**14.36**	**< 0.001**
No conditions	1				1			

#### Health-related quality of life-disutility score

Number of diagnosed conditions was a significant predictor of increased Disutility score (i.e. reduction in HRQOL) in both long- and short-haul drivers (Table 5), and reporting ≥3 conditions was associated with a 16% increase in Disutility score. Being overweight or obese was associated with a 3 and 2% increase respectively in Disutility score in short-haul drivers. Working ≤40 h/week and driving a rigid truck was associated with a reduction in Disutility score in short-haul drivers, whereas long-haul drivers working a shift type of “a single long trip between 2 locations” had reduced Disutility score. No other factors were significantly associated with Disutility score in either work type.

**Table 5:** Linear regression stratified by work type

Determinants of disutility	Long-haul drivers				Short-haul drivers			
	Exp(B)	95% CI		*p*	Exp(B)	95% CI		*p*
Age								
< 35 years	1.00	0.97	1.03	0.866	1.02	1.00	1.04	0.120
35–44 years	1.00	0.97	1.03	0.831	0.99	0.97	1.02	0.578
45–54 years	1.01	0.99	1.04	0.293	1.00	0.98	1.02	0.881
> 55 years	1.00				1.00			
Employment type								
Owner driver	1.00	0.96	1.03	0.805	0.98	0.96	1.01	0.255
Employee driver	1.00				1.00			
Work for > 1 company								
Yes	0.99	0.96	1.02	0.467	1.00	0.98	1.03	0.713
No	1.00				1.00			
Shift type								
Multiple trips between same base	0.99	0.96	1.01	0.334	0.99	0.97	1.02	0.510
Single long trip between 2 locations	0.97	0.95	0.99	**0.017**	0.99	0.96	1.03	0.663
Multiple trips between 2 locations	1.00				1.00			
Vehicle type								
Rigid truck and other	1.01	0.97	1.06	0.597	**0.97**	**0.96**	**0.99**	**0.005**
Road train	1.00	0.95	1.04	0.893	0.99	0.96	1.03	0.657
B Double	1.01	0.97	1.05	0.620	0.98	0.96	1.00	0.081
Articulated truck	1.00				1.00			
Payment type								
Other	1.02	0.98	1.07	0.376	1.02	0.98	1.05	0.320
Per trip/delivery	1.02	0.99	1.05	0.252	1.00	0.96	1.03	0.896
Single time pay	1.02	0.98	1.06	0.434	0.99	0.97	1.01	0.342
Km rate	1.00	0.98	1.03	0.753	1.03	0.98	1.08	0.294
Flat rate	1.00				1.00			
Working hours								
≤ 40 h	1.01	0.99	1.04	0.190	**0.98**	**0.95**	**1.00**	**0.038**
> 60 h	1.00	0.96	1.04	0.921	1.01	0.99	1.03	0.537
41–60 h	1.00				1.00			
BMI								
Obese	1.00	0.98	1.03	0.795	**1.03**	**1.01**	**1.05**	**0.015**
Overweight	1.01	0.97	1.04	0.720	**1.02**	**1.00**	**1.05**	**0.038**
Under or normal weight	1.00				1.00			
Diagnosed conditions								
1 condition	**1.06**	**1.04**	**1.09**	**< 0.001**	**1.06**	**1.03**	**1.08**	**< 0.001**
2 conditions	**1.11**	**1.08**	**1.15**	**< 0.001**	**1.08**	**1.05**	**1.10**	**< 0.001**
≥ 3 conditions	**1.16**	**1.13**	**1.19**	**< 0.001**	**1.16**	**1.14**	**1.19**	**< 0.001**
No conditions	1.00				1.00			

## Discussion

To our knowledge, this is the largest health-focused survey of Australian professional truck drivers to date. Our findings suggest that the physical health of truck drivers is poorer than average for the Australian population. Approximately 80% of truck drivers in our study were classified as overweight or obese compared to an average of 70% for Australian males [38]. Comparable prevalence rates of obesity in truck drivers have been reported by studies in the USA, Canada, UK and Australia [6, 19, 39–41]. The proportion of drivers reporting a diagnosis of three or more chronic health conditions was almost four times that of the Australian average, with back problems reported at nearly double the rate of the Australian population [24]. Truck drivers routinely experience factors that increase the risk of obesity (such as long working hours, limited opportunity to exercise and reduced access to health food options [10, 42]), as well as the risk of musculoskeletal conditions (such as high job demands, high job strain, low job control and sleep deprivation [43, 44]). The poor physical health profile demonstrated in our survey reflect many of the health risks drivers face at work.

The prevalence of diagnosed mental health conditions in our sample was consistent with the average Australian [24]. However, 1 in 2 drivers reported some level of psychological distress compared to an average of 1 in 3 for working age Australian men [45]. Suicide has been shown as one of the leading causes of death in young transport workers, second only to external causes of injury, such as motor vehicle crashes [13]. Regression analysis of our data revealed that younger drivers and those with multiple diagnosed medical conditions are at increased risk of severe psychological distress in both long- and short-haul drivers. The relationship between physical and mental health in drivers is important to address, as conditions like depression and anxiety have also been positively associated with the diagnosis of multisite musculoskeletal conditions [44].

In this study, long- and short-haul drivers displayed relatively minor differences in health profiles. While the distribution of diagnosed medical conditions was similar, a greater proportion of long-haul drivers reported dealing with chronic pain. Similar results were found in a Canadian study [46] where lifestyle factors like sleeping hours were associated with chronic pain in long-haul drivers. Other studies have identified frequent manual handling, seat discomfort and working night shifts as factors associated with chronic pain in long haul drivers [41, 47, 48]. In contrast, more short-haul drivers experienced severe psychological distress than long-haul drivers. This could be due to the large proportion of time spent in busy metropolitan traffic conditions and more frequent interaction with the general public on the roads [16]. Short-haul drivers in Australia identified other motorists as the source for most reported experiences of workplace violence [49] and listed the behaviour and presence of other road users as a top safety concern [50].

Our results suggest that younger drivers should be prioritised in interventions focused on improving mental health. This is particularly important when drivers tend not to access services for mental health in the early phase of an injury [15], if at all [51]. The findings of this study should also be used to guide implementation efforts, such as marketing mental health strategies through social media platforms, possibly led by industry regulators. While both long- and short-haul drivers would benefit from interventions aimed to reduce the incidence and impact of chronic health conditions, there are opportunities to tailor health-focused interventions to the nature of a driver’s work tasks. Short-haul drivers may benefit more from programs aimed at managing the stressors of frequent exposure to the public, whereas the focus for long-haul drivers should be on pain prevention and management strategies. Employers could also play a role in managing the stressors for short-haul drivers through developing the skills of supervisors in identifying and managing situations where drivers are at risk of stress [52]. A consequence of the absence of policy in this area is that we see the space being filled by community and industry-based organisations, which often leads to initiatives that are not fully evaluated so their effectiveness remains unclear. Strategies such as prioritising younger drivers in road safety actions plans or ensuring that health care providers integrate this knowledge into their management plan for truck drivers could provide a way forward in the prevention and management of truck driver ill health. Educating general practitioners and allied health on the different risk factors long- and short-haul drivers are exposed to would contribute to a preventative approach in the management of truck driver health and wellbeing. Regulators could also use this information in their guidance material and inspections in an effort to prevent these injuries.

Health and driving performance are linked as obesity, high number of diagnosed health conditions and poor mental health, all identified in this cohort of Australian drivers, have previously been associated with significant increased risk of preventable crashes [53–58]. Health is also a key predictor of work ability and despite the poor health profile described here, the greater majority of drivers rated themselves as having good or excellent work ability. This could suggest that other factors contributing to good work ability, like personal attitudes and work environment, compensate for the potential adverse effect of poor health on work ability [59]. Future studies focused on driver health should aim to measure more comprehensive work, lifestyle, personal and environmental factors to establish the impact of these factors on driver health.

### Strengths and limitations

This study presents findings from one of the largest health focused surveys of truck drivers in Australia. Our sample includes drivers across Australia who drive a variety of vehicles with various experience levels. However, there are some study limitations to be noted. While the recruitment strategy included a range of approaches, there is likely a bias towards drivers possessing the technological skills to easily access and complete the survey online. Despite this, the age distribution, male to female proportion and owner versus employee driver ratios are similar to workforce estimates [3]. The survey data relies on self-report and may be influenced by the narrative and memory of the drivers themselves, a common limitation of health-focused research [60] .

The application of validated measures such as the K6, reduced some impact of recall bias. As a cross-sectional survey, these results highlight areas for further investigation and cannot be used as a basis for establishing causal relationships. This study provides baseline data that establishes the health profile of Australian short- and long-haul truck drivers. This will add to the growing body of research into the health of truck drivers which will allow employers, regulators and government to make evidence-based decisions when designing and implementing interventions aimed at truck drivers.

## Conclusion

The health profile of Australian truck drivers appears to be worse than the general Australian population. Truck drivers are more likely to be overweight, report poor general health and be diagnosed with multiple chronic health conditions. Long haul drivers are more likely to experience chronic pain, whereas short-haul drivers are more likely to experience high levels of psychological distress. Across both driver types under the age of 35, the levels of severe psychological stress were higher than the national average. This study highlights the need for interventions targeted towards the prevention and management of mental and physical health conditions, in order to help drivers to be healthy and stay healthy at work.

## Supplementary Information

**Additional file 1.** Driving Health Online Survey. Online survey contents.

## Data Availability

The datasets generated and/or analysed during the current study are not publicly available due to confidentiality clause contained in the explanatory statement provided to participants. Only Monash University Driving Health Researchers with ethics approval can access the data. Aggregated and de-identified data are available from the corresponding author on reasonable request.
