# Supplementary material for: The physical and mental health of Australian truck drivers: a national cross-sectional study
Source: BMC Public Health. 2022 Mar 8;22:464. doi: 10.1186/s12889-022-12850-5 (PMC8903653; doi:10.1186/s12889-022-12850-5)
Supplement: Supplementary file 1 — Additional file 1. Driving Health Online Survey. Online survey contents. [file 12889_2022_12850_MOESM1_ESM.pdf]

DRIVING HEALTH

Welcome to the Driving Health Survey

Please read the study information in the Driving Health explanatory statement

Additional information about this study can be found on our drivinghealth.net.

Please indicate if you agree to participate in the research study under the following conditions:

- I have **read** the study information and understand the purpose and nature of this study.
- I am participating **voluntarily.**
- I understand that all information provided will be treated as **confidential**.

☐ Yes

☐ No

MONASH University

First, we need to know if you are eligible for this study.

1. In the last 12 months has your job included the transport of goods?

- ☐ Yes
- ☐ No

2. Do you drive a vehicle as the main part of your job?

- ☐ Yes
- ☐ No

3. What type of vehicle do you usually drive for your job?

- ☐ Car
- ☐ Van
- ☐ Rigid truck
- ☐ Flatbed truck
- ☐ Articulated truck
- ☐ B double
- ☐ Road train
- ☐ Other (please specify) ____
- ☐ Prefer not to say

We need to know more about your job

4. Do you own the vehicle you drive for work?

- ☐ Yes
- ☐ No
- ☐ Prefer not to say

5. Do you work for more than one company?

- ☐ Yes
- ☐ No
- ☐ Prefer not to say

6. Which of the following best describes a typical work shift for you?

- ☐ Multiple trips beginning and ending at a “home base” (i.e. warehouse, depot)
- ☐ A single long trip between two destinations
- ☐ Multiple trips between two destinations
- ☐ Prefer not to say

7. How far do you usually drive in one shift?

- ☐ 0-100 km of home base (i.e. warehouse, depot)
- ☐ Between 100-500km of home base (i.e. warehouse, depot)
- ☐ More than 500km from a home base (i.e. warehouse, depot)
- ☐ Prefer not to say

8. On average, how many shifts do you work per week? (write one number only)

____

9. On average, how many hours is a typical shift? (write one number only

____

10. How long have you been driving vehicles for a living?

- ☐ Less than 12 months
- ☐ 1-5 years
- ☐ 6-10 years
- ☐ 11-20 years
- ☐ 21-30 years
- ☐ More than 30 years
- ☐ Prefer not to say

11. During the last **month**, how were you usually paid?

- ☐ Flat hourly rate
- ☐ Flat daily rate
- ☐ Single time pay plus overtime
- ☐ Flat weekly rate
- ☐ Trip rate/per fare
- ☐ Rate based on km travelled
- ☐ Per delivery
- ☐ Other (please specify)

____

- ☐ Prefer not to say

Transport workers experience distinct health risks. The next few questions are about your physical and mental health - your answers will help us improve the working health of Australian drivers.

12. Under each heading, please tick the ONE box that best describes your health TODAY.

MOBILITY

I have no problems with walking around ❑

I have slight problems with walking around ❑

I have moderate problems with walking around ❑

I have severe problems with walking around ❑

I am unable to walk around ❑

PERSONAL CARE

I have no problems with washing or dressing myself ❑

I have slight problems with washing or dressing myself ❑

I have moderate problems with washing or dressing myself ❑

I have severe problems with washing or dressing myself ❑

I am unable to wash or dress myself ❑

USUAL ACTIVITIES *(e.g. work, study, housework, family or leisure activities)*

I have no problems doing my usual activities ❑

I have slight problems doing my usual activities ❑

I have moderate problems doing my usual activities ❑

I have severe problems doing my usual activities ❑

I am unable to do my usual activities ❑

PAIN / DISCOMFORT

I have no pain or discomfort ❑

I have slight pain or discomfort ❑

I have moderate pain or discomfort ❑

I have severe pain or discomfort ❑

I have extreme pain or discomfort ❑

ANXIETY / DEPRESSION

I am not anxious or depressed ❑

I am slightly anxious or depressed ❑

I am moderately anxious or depressed ❑

I am severely anxious or depressed ❑

I am extremely anxious or depressed ❑

- We would like to know how good or bad your health is TODAY.
- This scale is numbered from 0 to 100.
- 100 means the best health you can imagine.
  0 means the worst health you can imagine.
- Mark an X on the scale to indicate how your health is TODAY.
- Now, please write the number you marked on the scale in the box below.

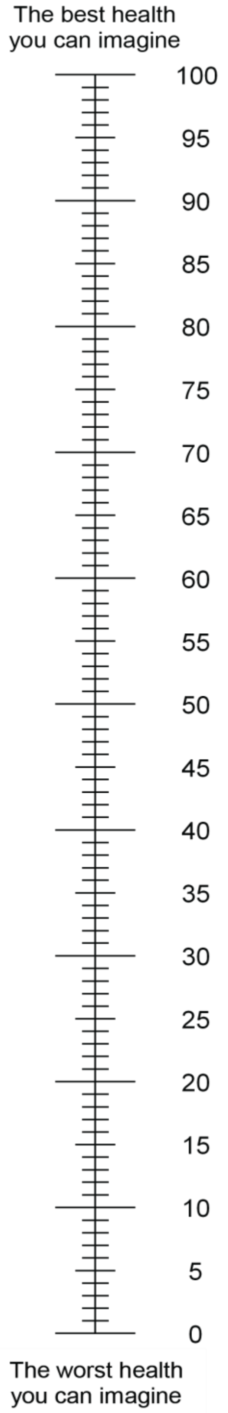

YOUR HEALTH TODAY = [ ]

MONASH University

13. Over the past **12 months** have you had treatment for, or been told by a doctor or a nurse that you have any of the following health conditions? (please select all that apply)

- ☐ Asthma
- ☐ Cardiovascular disease (e.g. heart attack, chest pain, heart failure)
- ☐ Diabetes
- ☐ Chronic Obstructive Pulmonary Disease (COPD)
- ☐ Arthritis
- ☐ Cancer
- ☐ Osteoporosis or osteopenia
- ☐ Stroke or cerebrovascular disease
- ☐ None of these
- ☐ Prefer not to say

14. Over the past **12 months** have you had treatment for, or been told by a doctor or a nurse that you have any of the following health conditions? (please select all that apply)

- ☐ High blood pressure or hypertension
- ☐ Mental health problems (e.g. anxiety, depression, panic attacks, pos- traumatic stress disorder etc)
- ☐ Sleep apnoea
- ☐ Migraines or severe headaches
- ☐ Traumatic injury or accident
- ☐ Hayfever or allergic rhinitis
- ☐ Back problems
- ☐ High cholesterol
- ☐ None of these
- ☐ Prefer not to say

15. Have you experienced any physical pain in the last **week**?

- ☐ Yes – continue to question 16
- ☐ No – continue to question 18

16. Using a scale of 0 to 10, with **0** being **no pain at all** and **10** the **worst possible pain**, which best describes the pain you have felt during the **past week** (please circle your answer).

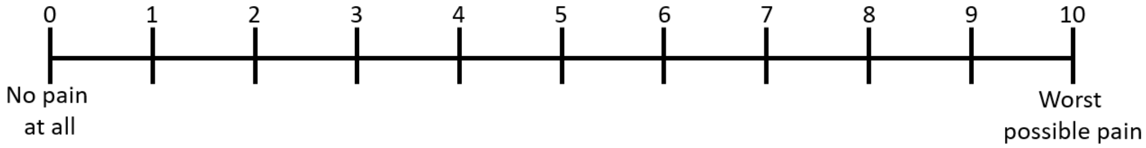

17. How long have you had your current pain problem?

- ☐ Less than one week
- ☐ 1 - 2 weeks
- ☐ 3 - 4 weeks
- ☐ 4 - 5 weeks
- ☐ 6 - 8 weeks
- ☐ 3 - 6 months
- ☐ 6 - 9 months
- ☐ 9 - 12 months
- ☐ Over 1 year
- ☐ Prefer not to say

The following questions ask about how you have been feeling during the past 4 weeks. For each question, please choose the option that best describes how often you had this feeling.

18. In the last 4 weeks, how often did you feel……

	All of the time	Most of the time	Some of the time	A little of the time	None of the time
….nervous?	☐	☐	☐	☐	☐
….hopeless?	☐	☐	☐	☐	☐
….restless or fidgety?	☐	☐	☐	☐	☐
….so sad nothing could cheer you up?	☐	☐	☐	☐	☐
….everything was an effort?	☐	☐	☐	☐	☐
….worthless?	☐	☐	☐	☐	☐

19. Using the scale of 0 to 10, with **0** being **completely unable to work** and **10** being **able to work at your best**, which best describes your ability to work today (please circle your answer)?

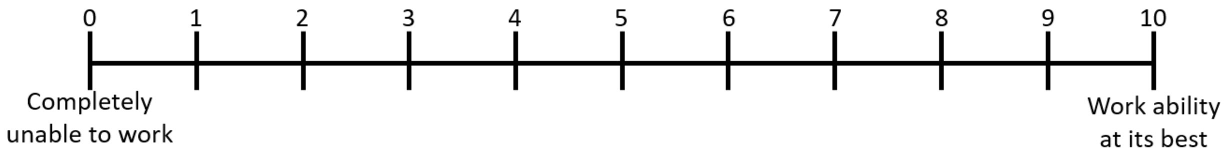

20. In general would you say your health is:

- ☐ Excellent
- ☐ Very Good
- ☐ Good
- ☐ Fair
- ☐ Poor
- ☐ Prefer not to say

MONASH University

Please tell us your height and weight. If you are not sure, please give your best guess.

21. What is your current height (in cm)?

____

22. What is your current weight (in kg)?

____

Next, we would like to know about your driving

23. In the last **12 months**, what is the total number of crashes you have been involved in while driving for work purposes?

- ☐ 0
- ☐ 1
- ☐ 2
- ☐ 3
- ☐ 4
- ☐ 5 or more
- ☐ Prefer not to say

24. Over the past **month**, what is the total number of near misses you have been involved in while driving for work purposes?

- ☐ 0-5 (once a week)
- ☐ 6-10
- ☐ 10-20
- ☐ 20-30 (daily)
- ☐ 30-50 (more than once a day)
- ☐ Prefer not to say

To make full sense of how your work affects your health, we need to know a little bit about you.

25. How old are you?

- ☐ <18
- ☐ 18-24
- ☐ 25-34
- ☐ 35-44
- ☐ 45-54
- ☐ 55-64
- ☐ >65
- ☐ Prefer not to say

26. Are you:

- ☐ Male
- ☐ Female
- ☐ Prefer not to say

27. Please tell us how you heard about the Driving Health study

- ☐ I was sent a text
- ☐ Saw it on Facebook
- ☐ Email from my employer
- ☐ Heard about it on the radio
- ☐ Heard from a friend/colleague
- ☐ Found it on the web
- ☐ Other (please specify) ____________________

MONASH University

Thank you for completing this survey!

Would you like to further contribute to improving the workplace health of the Australian Driving Community?

We need to know more about the factors that influence your health and would like to ask you some questions on the phone, which we expect will take around 25 minutes to complete.

The first 700 people who complete the second survey will receive a $20 Coles voucher.

Would you like to participate in the next part of the study?

- ☐ Yes
- ☐ No

If you would like to participate in the next part of our study please provide your contact details below.

This information will only be used by us to contact you for this research purpose and will not be made available to anyone else.

- ☐ First name ____
- ☐ Surname ____
- ☐ Best contact number ____

Preferred contact times (when you will not be busy driving)

- ☐ Weekdays before 5PM
- ☐ Weekdays after 5PM
- ☐ Weekends
- ☐ Any other/specific time? ____

MONASH University
